# Mentor-Mentee relationship in Medicine: How to develop trust?

**DOI:** 10.12669/pjms.40.11.9372

**Published:** 2024-12

**Authors:** Khadijah Mukhtar, Mahnoor Mukhtar, Syed Abdullah Haider, Sadia Iram

**Affiliations:** 1 Khadijah Mukhtar, University College of Medicine and Dentistry (UCMD), Lahore, Pakistan; 2 Mahnoor Mukhtar, University College of Medicine and Dentistry (UCMD), Lahore, Pakistan; 3 Syed Abdullah Haider, University College of Medicine and Dentistry (UCMD), Lahore, Pakistan; 4 Sadia Iram Lahore Medical and Dental College, Lahore, Pakistan

**Keywords:** Mentor mentee relationship, Trust, Importance of communication, Feedback, Empathy, Respect

## Abstract

A mentor-mentee relationship, also known as a mentoring relationship, is a professional or personal partnership in which a more experienced or knowledgeable individual (the mentor) provides guidance, support, and advice to a less experienced or knowledgeable individual (the mentee). The primary purpose of this relationship is to facilitate the mentee’s personal and professional growth. Mentors typically share their expertise, wisdom, and experiences to help mentees navigate their career or personal development paths. In this qualitative exploratory study 10 mentees were interviewed and analysis was done based on their answers to find out the strengthening factors of a mentor-mentee relationship particularly focusing on the qualities and traits required to build trust. The results of this research indicate that trust among mentors and mentees could be developed by respect, confidentiality, empathy, humbleness, hard work and good communication from both sides.

## INTRODUCTION

Mentorship is experience and/or knowledge-based guidance. A mentor-mentee relationship is a mutually beneficial partnership between an experienced individual (the mentor) and a less experienced person (the mentee/proteges).^1^ The mentor serves as a guide, offering support, advice, and insights based on their knowledge and expertise. They help the mentee navigate their personal or professional journey, sharing wisdom, providing constructive feedback, helping them set and achieve goals and have positive effects on competence and mental well-being.^2^ This relationship is valuable because it accelerates the mentee’s growth and development, boosts confidence, and fosters a sense of belonging and support. Early-career researchers (ECRs), in particular, can benefit from mentorship to navigate challenges in academic and non-academic life and careers.^3^

Trust is developed on the pillars of transparent communication, dependability, and mutual respect. To nurture trust, it is essential for both the mentor and mentee to engage in two-way listening, exchange experiences and insights, and communicate openly about expectations and limitations.^4^ The mentor assumes the responsibility of offering guidance and support. Conversely, the mentee is expected to embrace feedback, take ownership of their goals, and display a willingness to learn and adapt. Through consistent participation in transparent and respectful interactions, trust naturally deepens, fostering a secure and supportive environment conducive to learning and personal development.^5^ A pivotal determinant of trust lies in how well the mentor respects the mentee and their beliefs, as underscored by Krishna’s Ring Theory of Personhood, which highlights the impact of personal beliefs on professional identity and mentoring perceptions.^6^

The mentor-mentee relationship within global medical mentoring programs has historical ties to the traditional apprenticeship model, wherein experienced practitioners guided the training of aspiring physicians.^7^ Adapting to the intricacies of contemporary healthcare, structured mentorship programs have arisen to meet the demand for comprehensive support in medical education. Beyond the transmission of clinical knowledge and skills, these relationships assume a crucial role in delivering emotional support, career guidance, and cultivating a culture of continuous learning. Worldwide, medical mentorship programs aim to facilitate the growth of competent and compassionate healthcare professionals by fostering these vital one-on-one connections. In Pakistan, University College of Medicine and Dentistry is one of pioneers of mentoring programmes and has been continuously running a successful mentoring program for the last six years.^8^

After a deliberate analysis of the previous research done on studying the different dynamics of mentor-mentee relationship, not much data was found on the strategies to build trust between the mentors and the mentees. The main objective of this study was to explore the factors that help develop trust between the two.

Like any other relationship, trust is an important component of teacher and student relationship. Degree to which students rely on teachers for their academics depends upon the trust level maintained by the teachers. The way of teaching and the way teachers used to treat students influence student’s attempts to study well in any institutional environment.^9^ Trust has motivational power that brings positive psychological and emotional changes especially to those facing difficult times in their life. Trust in a relationship plays an important role in the quality of education and grasping of knowledge.^10^ For the success of a mentoring program, mentors should have the quality of assisting their students and being their role models.^11^

## METHODS

Qualitative exploratory study was performed at University College of Medicine and Dentistry (UCMD), Lahore, Pakistan over the period of one month in December 2023. One to one interview was conducted with 10 mentees who had two-year experience of mentoring sessions with their respective mentors. The mentors were well trained by attending at least three consecutive workshops conducted by faculty development program. Mentorship was the part of formal mentoring program at UCMD from 2016. Convenient purposive sampling was done and mentees from fourth and final MBBS were included in this study for interviews. Mentees were provided with participant’s information sheets that included interview questions and consent form. Interview questions were developed by research team through extensive literature search which were then sent to two renowned medical educationists for expert validation. Interview questions were pre-validated by the experts. After having verbal and written consent, open–ended questions were asked from all study participants. The question answer session was audio recorded with consent for recording. After the interview, data was transcribed by Otter.ai and transcribes data were sent to respective participant to ensure dependability. Later data an

### Ethical Approval:

The study was approved by the institutional ethics committee (Ref. No.: ERC122/23/12; dated: December 1, 2023).

## RESULTS

Data analysis was performed through qualitative data analysis tools by using Atlas-Ti and themes along with respective quotations were carved out. The factors that develop trust between mentors and mentees are listed in Table-I.

**Table-I:** Themes and respective quotations.

Themes	Quotations
Respect	“In my opinion, in order to develop strong relationships, giving and gaining respect is so important.”
Confidentiality	“Well, I know from the beginning that whenever my mentor tells my class-fellows and my teachers about the things I used to share with him, that will be our last session together.”
Empathy	“When you ask for help from your mentor then they help you without any other benefits, without other monetary benefits, they completely devote their time, their efforts towards making you a better individual.”
Humbleness	“Trust is if a student has any issue with studies, he should have courage to discuss that issue with his teacher and the teacher will also discuss that problem with patience.”
Hard-work	“If the teacher doesn’t make an effort in solving our issues that leads to distrusting a teacher.”
Communication	“Mentor should be more open; he should be more welcoming because there are some teachers you are scared of going to them”.

In our study, the researchers were keen to find out the factors to develop trust amongst mentors and mentees. As, at UCMD there were formal mentoring program, we conducted one to one interview with mentees. While, thematic analysis it was found that relationship will trustworthy between mentors and mentees if mentor has good listening skills along with the confound communication skills. Proper and good communication skills of mentors, allow mentees to express their feelings and discuss problems with mentors more. Even confidentiality also have an effect on building trust as it allows mentees to express themselves more openly. Our study also showed that respect, humbleness, empathy and hard-work are the key characteristics of the mentors in building trust with mentees.

## DISCUSSION

Establishing trust between a mentor and a mentee is crucial for promoting a constructive and successful mentoring relationship. This study maps out the factors that help in building trust amongst mentors and mentees, thus, the mentor can provide tailored guidance and accelerating the mentee’s learning curve.

A positive mentoring relationship can produce significant benefits for the mentee in terms of academic research productivity and career satisfaction.^12^ Achieving success in this collaborative effort requires mutual dedication from both parties. The mentee’s commitment to acquiring knowledge is pivotal in eliciting the best efforts from the mentor. This study underscores that mentors can readily cultivate trust by addressing the factors outlined in Table-I. Essential qualities for mentors include the ability to provide guidance selflessly and offering both intellectual and emotional support. Factors identified by students as conducive to building trust in mentor-mentee relationships encompass respect, confidentiality, empathy, humility, diligence, and effective communication. A mentor is expected to offer steadfast guidance, provide constructive feedback, openly discuss student challenges, share strengths, and actively forge strong connections with students. Our findings align with prior research by Frei and Stamm, emphasising the crucial role of mentoring in students’ career counselling.^13^

In this study, it shows that respect is the cornerstone of mentor mentee relationship. In literature, research has shown that when tutors exhibit respect, it promotes the mentee’s self-viability and expert development.^14^ Respectful connections can incorporate esteeming the mentee’s viewpoints, giving valuable input, and perceiving their commitments. A study by Steinert et al. emphasises that respectful conduct from mentors contributes to a positive learning environment and fosters mutual trust.^15^

Our study shows, confidentiality is another critical aspect of building trust among mentor and mentee. In mentor-mentee relationship, mentors should be very conscious about the information shared by mentee, including academic, financial, social and personal challenges and struggles. Maintaining confidentiality promises mentees that their worries and proficiencies will be kept private, so nurturing an environment where they feel safe to share openly.^16^ The quickest way to develop trust between two people is by keeping confidentiality.^17^ Complete confidentiality must be upheld to ensure that mentees can freely express their honest opinions and provide feedback on teaching methodologies without the fear of facing any repercussions or holding grudges against them.

The cornerstone of an effective mentoring often shoots from the hard work devoted by a mentor who donates energy, time, and effort to build a strong relationship with mentee. 18 A study by Chen, it showed that hard-work is a price that mentors and mentees both should apply to build trust and achieve their professional and personal goals.^19^

Our research study showed the significance of effective communication within mentor-mentee relationships. Given the expansive and influential role of communication in educational programs, it is imperative to thoroughly examine and acknowledge its various facets. A comprehensive communication approach between a mentor and mentee holds the potential to instil enthusiasm and other qualities in the mentee, thereby fostering a stronger and more trustworthy relationship.^20^

Humbleness and empathy play a pivotal role in maintaining a healthy and productive mentor-mentee relationship. It serves as a two-way channel that fosters growth and understanding between mentor and mentee.^21^ For mentors, Empathy allows them to assess the mentee’s personal relationships, identify areas for improvement, and tailor their guidance to meet the mentee’s specific needs. Mentees, on the other hand, benefit from feedback by gaining insights into their strengths and weaknesses, which aids in personal and professional development.^22^

Unrealistic expectations for mentors or mentees can be a real stressor. Occasionally, mentors and mentees may expect too much from one another. One must realise that the other person may be busy with work–life challenges. Mentees should be understanding and appreciative of the benefits of the relationship.^3^ Expectations from both sides should be communicated and addressed in the beginning to avoid any conflicts or issues later. All these steps ensure a good mentor-mentee relationship providing space for growth to both sides.

### Limitation:

This study was done only by taking mentees perception. In future studies Mentors perception should also be taken into account.

## CONCLUSION

In medicine, a mentee who trusts their mentor is more likely to openly discuss challenges, seek guidance, and readily accept constructive feedback. This trust fosters a positive learning environment where the mentee feels safe to explore new skills and ideas. This research concludes that in order to develop a good mentor-mentee relationship, trust is the major component further elaborating that trust among mentors and mentees can be developed by respect, confidentiality, empathy, humbleness, hard work and good communication.

### Authors Contributions:

**KM:** Conception of work and acquisition of data.

**MM:** Analysis, interpretation of data and literature search.

**SAH:** Acquisition of data, composition of the article and added references.

**SI:** Transcription of interviews, composition of the article, literature search and responsible for the accuracy of study
